# Characteristics and Management of Candidaemia Episodes in an Established *Candida auris* Outbreak

**DOI:** 10.3390/antibiotics9090558

**Published:** 2020-08-30

**Authors:** Juan V. Mulet Bayona, Nuria Tormo Palop, Carme Salvador García, Paz Herrero Rodríguez, Vicente Abril López de Medrano, Carolina Ferrer Gómez, Concepción Gimeno Cardona

**Affiliations:** 1 Microbiology Department, Consorcio Hospital General Universitario de Valencia, 46014 Valencia, Spain; tormo_nur@gva.es (N.T.P.); salvador_cargar@gva.es (C.S.G.); concepcion.gimeno@uv.es (C.G.C.); 2 Infectious Diseases Department, Consorcio Hospital General Universitario de Valencia, 46014 Valencia, Spain; paz.herrero@goumh.umh.es (P.H.R.); abril_viclop@gva.es (V.A.L.d.M.); 3 Anestesiology and Reanimation Department, Consorcio Hospital General Universitario de Valencia, 46014 Valencia Spain; ferrer_cargoma@gva.es; 4 Microbiology Department, University of Valencia, 46100 Burjassot, Spain

**Keywords:** *Candida auris*, candidaemia, fungi, yeast, outbreak, multidrug-resistant, colonisation, surveillance

## Abstract

The multi-resistant yeast *Candida auris* has become a global public health threat because of its ease to persist and spread in clinical environments, especially in intensive care units. One of the most severe manifestations of invasive candidiasis is candidaemia, whose epidemiology has evolved to more resistant non-*albicans* *Candida* species, such as *C. auris*. It is crucial to establish infection control policies in order to control an outbreak due to nosocomial pathogens, including the implementation of screening colonisation studies. We describe here our experience in managing a *C. auris* outbreak lasting more than two and a half years which, despite our efforts in establishing control measures and surveillance, is still ongoing. A total of 287 colonised patients and 47 blood stream infections (candidaemia) have been detected to date. The epidemiology of those patients with candidaemia and the susceptibility of *C. auris* isolates are also reported. Thirty-five patients with candidaemia (74.5%) were also previously colonised. Forty-three patients (91.5%) were hospitalised (61.7%) or had been hospitalised (29.8%) in the ICU before developing candidaemia. Antifungal therapy for candidaemia consisted of echinocandins in monotherapy or in combination with amphotericin B or isavuconazole. The most common underlying disease was abdominal surgery (29.8%). The thirty-day mortality rate was 23.4% and two cases of endophtalmitis due to *C. auris* were found. All isolates were resistant to fluconazole and susceptible to echinocandins and amphotericin B. One isolate became resistant to echinocandins two months after the first isolate. Although there are no established clinical breakpoints, minimum inhibitory concentrations for isavuconazole were low (≤ 1 μg/mL).

## 1. Introduction

An increase in the prevalence of *Candida* bloodstream infections (candidaemia) and a shift in the epidemiology have been observed in recent years, especially since the emergence of the multidrug-resistant yeast *Candida auris* [1]. *C. auris* was first identified in 2009, from the auditory canal of a Japanese patient [2] and, since then, it has been reported in a large variety of body parts and in the six continents of the world [3]. This yeast is considered a growing menace to global health for several reasons, which include its resistance to multiple commonly used antifungals, its problematic identification in the laboratory and its facility to spread among patients, causing nosocomial outbreaks, especially in intensive care units (ICU) [4]. Different organisations, such as Centers for Disease Control and Prevention (CDC) or the European Centre for Disease Prevention and Control (ECDC), claim that there is an emergency in using reliable methods to identify *Candida* spp. isolates to the species level and encourage hospitals to do screening colonisation studies in order to control the outbreaks caused by *C. auris* [5,6]. In our setting, there is an ongoing *C. auris* outbreak since October 2017 [7], and, despite the implementation of different control measures (e.g., periodic screening for *C. auris* surveillance), we still report cases, showing the difficulty in eradicating this nosocomial pathogen.

The aim of this study is, first, to describe the *C. auris* outbreak that is currently ongoing in our setting and the measures established in order to control it. Secondly, we analyse the evolution in the *Candida* species distribution causing candidaemia in our setting since 2011 and the clinical and epidemiological characteristics of all patients diagnosed with *C. auris* candidaemia, as well as the antifungal susceptibility of the isolates.

## 2. Results

### 2.1. Description of the Outbreak

In September 2017, *C. auris* was detected for the first time in our hospital in the urine culture of a patient. One month later, in October 2017, a second case was detected in a blood culture of another patient, being therefore the first case of candidaemia. Since then, screening for *C. auris* colonisation is part of the infection control practices in the ICU, where the outbreak is located. Screening is performed before a patient is admitted in the ICU and once a week until hospital discharge. When a colonised or infected patient is discharged to a general ward, surveillance is also performed, and when a case is detected outside the ICU, surveillance cultures are performed to contacts in the ward. Together with the screening of patients, other measures were implemented, such as the isolation of cases, patient cohorting, the reduction of health workers in contact with colonised patients, the decolonisation of patients with clorhexidine solutions and environmental cleaning with hydrogen peroxide solutions (Oxivir^®^). Environmental surveillance is also performed in the ICU once a year or when an increase in the number of cases is produced, and *C. auris* has been isolated from mattresses. tables, computers and an emergency button. The entire ICU was also cleaned with a more concentrated hydrogen peroxide solution following the environmental cultures.

**Figure 1:**
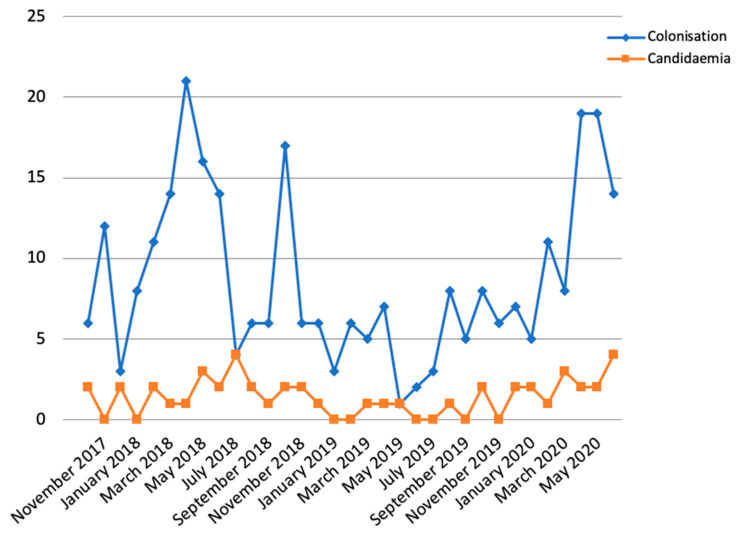
New colonised patients and candidaemia episodes from October 2017 to June 2020.

A total of 287 colonised patients and 47 candidaemia episodes (35 of them were also previously colonised) have been detected since the beginning of the outbreak (Figure 1). Our ICU includes 31 beds and it is divided in two sections: a general ICU and a cardiac ICU. Due to the large amount of cases and the long evolution of the outbreak, the *C. auris* outbreak already affects the entire ICU. In July 2018, despite the implementation of infection control practices and surveillance cultures, an increase was observed with four new patients with candidaemia (Figure 1). After that, new episodes of candidaemia continued to be detected but at a lower frequency. However, in April 2020, the incidence of colonisation and candidaemia increased again, coinciding with the novel coronavirus SARS-CoV-2 pandemic in Spain (Figure 1). Recently, a PCR assay has been introduced in our laboratory for the detection of *C. auris* in surveillance samples, which is expected to reduce the response time from 24–48 h to 1 h, compared to conventional culture (unpublished data).

### 2.2. Characteristics of C. auris Candidaemias

**Figure 2:**
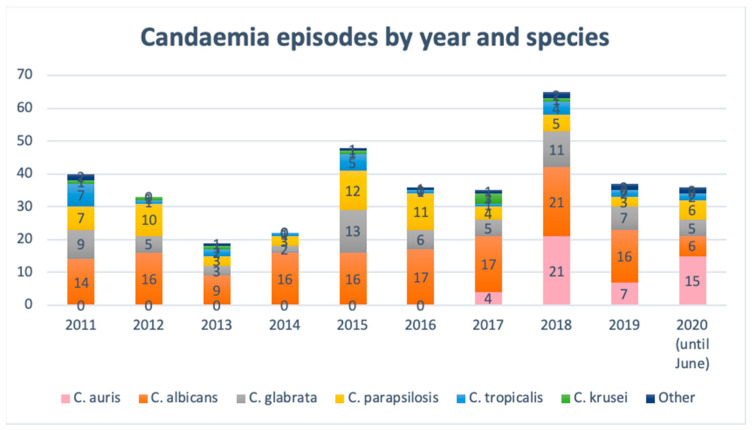
Species distribution in candidaemias produced in our setting since 2011 to June 2020.

The evolution of species distribution causing candidaemia since 2011 in our setting is shown in Figure 2. Although *C. albicans* has always been the most isolated species, a high proportion of more resistant Candida species (e.g., *C. glabrata*, *C. parapsilosis*) are also usually isolated in all studied years. In 2017, when *C. auris* emerged, this pathogen rapidly spread and caused several episodes of candidaemia. Especially remarkable is the large increase in candidaemia in 2018, the first year since the outbreak was established, becoming the year with most candidaemia episodes reported. In the first half of 2020, a large number of candidaemia episodes have also been produced, especially due to *C. auris*.

The median length of hospital stay before the candidaemia episode was 29 days (IQR 19.5–39.5). The median total length of hospital stay was 70 days (IQR 39.5–85). A total of 91.5% of patients (43/47) were hospitalised or had been hospitalised in the ICU before developing candidaemia. In 29 patients (61.7%), candidaemia was detected when the patient was in the ICU, while in 14 patients (29.8%), candidaemia was detected once the patient was discharged from the ICU, to a general ward. Four patients admitted to a general ward (8.5%) were never hospitalised in the ICU but developed *C. auris* candidaemia. However, those cases could not be related to other cases produced in a general ward. The median length of ICU stay before the candidaemia episode was 28 days (IQR: 19.5–40.5) and the median total length of the ICU stay was 29 days (IQR: 16–49.5). Only one patient developed candidaemia in the first week of stay in the ICU, five patients in the second week and nine patients in the third week. The remaining 28 patients took four or more weeks since admission to the ICU until the candidaemia episode. Epidemiological and clinical characteristics, antifungal treatment and outcome of 47 patients with *C. auris* candidaemia are summarised in Table 1. In seven patients, *C. auris* was isolated along with another *Candida* species: three were mixed with *Candida albicans*, two with *Candida tropicalis*, one with *Candida krusei*, and one with *C. albicans* and *Candida parapsilosis*. Antifungal treatment was administered until two weeks after a negative blood culture, and it consisted of echinocandins in monotherapy (46.8%) or in combination with amphotericin B (25.5%) or isavuconazole (21.3%), when this most recent azole was available in our hospital. The median duration of the antifungal treatment was 21 days (IQR: 15–25).

Surveillance cultures were conducted in all patients except four: the first case and three patients because they were not hospitalised in the ICU. From the performed cultures, 35 were positive (81.4%), 9 for both axillary-rectal and pharyngeal samples (25.7%), 24 only for the axillary-rectal sample (68.6%) and two only for the pharyngeal sample (5.7%). The median time since admittance to the ICU to a positive colonisation sample was 14 days (IQR 9.5–19.5). Six patients had a positive colonisation sample in the first week since they were admitted to the ICU (17.1%), one of which was already hospitalised in a general ward. Thirteen patients were positive to colonisation in the second week after they were admitted to the ICU (37.1%), two of which were already hospitalised in a general ward. Eight patients were positive to colonisation in the third week (22.9%) and the remaining eight patients were positive in the fourth or more weeks after their admittance to the ICU (22.9%), most of which were already hospitalised in a general ward. One patient was positive at the admission screening in the ICU, but this patient had a prior stay in the ICU registered from a month before. The median time since a positive colonisation sample to the candidaemia episode was 16 days (IQR 8–25).

Antifungal susceptibility of *C. auris* isolates is reported in Table 2. Only the first isolate of every patient was included in the table, as susceptibility did not differ greatly. However, it is noteworthy that an isolate that was resistant to echinocandins was isolated from a patient from whom a susceptible strain was isolated two months earlier. This patient presented a first episode of candidaemia associated with a permanent central venous catheter and was treated with anidulafungin for 28 days and antifungal lock of the catheter with anidulafungin until the catheter was replaced, but a second episode of candidaemia with the resistant strain was produced.

## 3. Discussion

*C. auris* is a global public health threat because of its ease to persist and spread in a clinical environment, among other reasons. During an outbreak, patients are colonised with *C. auris* at several body sites, including axilla, groin, nostrils, ears and rectum, and it also has been detected in beds, tables, floors, walls, equipment and monitors [8,9,10,11,12]. Several measures were established in order to control the outbreak, following the recommendations of the ECDC [6], the CDC [5] and the previous experience of other established outbreaks, as the first European outbreak described in a London cardio-thoracic centre [13] or the first Spanish outbreak described in [11]. These infection control practices include the strict isolation/cohorting of cases, decolonisation with chlorhexidine, regular environmental cleaning and the implementation of screening colonisation studies in the high-risk hospital environments such as the ICU. Axilla and groin swabs are usually recommended for screening, although other body parts can be sampled if it is clinically relevant [6]. In our setting, axillary-rectal and pharyngeal swabs were selected for the screening and showed good performances, especially axillary-rectal swab (76.7% of tested patients with candidaemia were previously positive for this sample). All these measures allowed a reduction in the frequency of the isolation of *C. auris* in both clinical samples and colonisation samples. However, two years after the outbreak started, and despite our efforts at establishing control measures and epidemiological surveillance, *C. auris* is still being isolated from colonisation samples and, what is most worrying, from blood cultures, showing that this nosocomial pathogen is very difficult to eradicate and can cause important infections.

Two peaks are observed in the evolution of the outbreak. The first one was in 2018, corresponding to the first months of the outbreak, and it could be in part because the control measures were not strictly followed by all health workers, especially in the holiday period, with some replacement staff. The second peak was in April and May 2020, coinciding with the peak of the SARS-CoV-2 pandemic in Spain. Hospitalisation rooms had to be reorganised in order to isolate patients affected by COVID-19 and that forced ignoring other nosocomial pathogens. Relaxing the control measures for *C. auris* could explain the increase in the cases (both colonisation and candidaemia) in those two periods, reinforcing the importance of infection control practices. It is important to note that the higher peak in candidaemia (July 2018) overlaps the lower peak in colonisations. This is because the patients are first colonised and approximately two weeks after develop candidaemia (the median time since ICU admittance to colonisation is 14 days and since admittance to the candidaemia episode is 28 days); therefore, a peak in colonisations is expected to produce an increase in candidaemia later.

Candidaemia is a life-threating condition in critically ill patients, which makes it crucial to understand local epidemiologic trends and the antifungal susceptibility of etiological agents. Multiple studies have shown an increase in the incidence of candidaemia and a shift to uncommon Candida species in recent years [1,14,15,16], including multidrug-resistant species such as *C. auris*, which has further complicated their management. In our setting, *C. albicans* is usually the predominant species, causing between a third and a half of candidaemia, followed by *C. glabrata* and *C. parapsilosis* alternating in second place until 2017, similar to other data reported in Spanish hospitals [17]. Although a clear tendency in species distribution over the years cannot be observed, what is clear is that candidaemia increased greatly in the first year of the outbreak, and *C. auris* has displaced *C. glabrata* and *C. parapsilosis* in our hospital, becoming the most isolated species in 2018, equal to *C. albicans*, and even exceeding *C. albicans* in the first half of 2020. It is noteworthy that in seven patients, *C. auris* was isolated along with another *Candida* species, which is a higher proportion than mixed non-*C. auris* candidaemia in the same period, which totalled five cases. Most mixed candidaemia was produced in 2018, when the larger increase in *C. auris* candidaemia was produced.

Several known risk factors for developing candidaemia [18,19] were present in a high number of our patients affected with *C. auris* candidaemia, like prior antibiotic exposure (91.5%), central venous catheter (83.0%), mechanical ventilation (61.7%), previous surgery (51.1%), previous colonisation with *C. auris* (74.5%) and ICU stay more than two weeks (70.2%). Gastrointestinal disease, both surgical and non-surgical, was the most common underlying disease (48.9%), which is also common for other *Candida* species candidaemia. A total of 14 patients with prophylactic antifungal treatment, 11 of them with echinocandins, developed candidaemia. Previous exposure to fluconazole or echinocandins has been associated with a higher incidence in *C. auris* candidaemia, which could be due to a selective pressure for *C. auris* [20]. Interestingly, age, APACHE II and Charlson comorbidity index were relatively low in the patients affected by *C. auris* candidaemia, which is also reported in a study from India comparing *C. auris* with non-*auris Candida* candidaemia [20]. This could be attributed to the profiles of patients that are usually admitted in the ICU, where the outbreak is established. As a complication of candidaemia, and despite the antifungal treatment, we detected two cases of endophtalmitis, which is less common than for other *Candida* species [21].

The crude mortality rate of *C. auris* candidaemia at 30 days in our series was 23.4%, similar to that reported by other recent studies [22,23,24]. However, this is lower than the mortality rates reported in initial studies of *C. auris* candidaemia [25,26,27], ranging 30–60% approximately, although *C. auris*-attributable mortality cannot be calculated from those series. The attributable mortality rate for *C. auris* candidaemia is difficult to evaluate because it affects critically ill patients with multiple comorbidities, though it should be analysed in futures studies.

*C. auris* is a multidrug-resistant yeast, and levels of resistance can vary between isolates, so antifungal susceptibility testing must be performed. There are no established susceptibility breakpoints, although CDC reported tentative breakpoints [28]. According to those breakpoints, all isolates were resistant to fluconazole and susceptible to echinocandins and amphotericin B. However, an isolate that was resistant to echinocandins was detected in one patient two months after the initial isolation of a sensitive strain. This might be due to a prolonged treatment with anidulafungin in the treatment of the first candidaemia episode, which exerted antimicrobial pressure. Resistance to echinocandins, amphotericin B and even pan-resistant isolates have been reported, in some cases after a prolonged treatment with these antifungals [29,30]. This demonstrates the need for continued surveillance, even in serial isolates from the same patient, and encourages being cautious when prescribing antifungal drugs. However, in our series, despite the intensive use of echinocandins, either in monotherapy or in combination, and also as an empirical treatment, no other strain developed resistance. MICs for posaconazole and voriconazole were variable, although the high resistance to fluconazole and to other azoles reported in the literature makes them not recommended to treat *C. auris* infections [31]. All tested isolates showed low MICs for isavuconazole (≤1 μg/mL), although there is limited evidence of the effectiveness.

The reported outbreak in our setting is one of the most important globally, with prolonged transmission over two and a half years. Although genotypic typing of the strains is still ongoing, epidemiological tracing is highly consistent with the existence of an outbreak (same hospital ward). Preliminary results with some strains suggest they belong to the South African clade, the same as other Spanish isolates [11].

## 4. Materials and Methods

Consorcio Hospital General Universitario de Valencia (CHGUV) is a 503-bed tertiary hospital which provides medical assistance to a population of around 400,000 people in Valencia, Spain. The evolution of the *C. auris* outbreak which started in October 2017 in our setting is described, as well as the infection control practices established. A screening colonisation study is performed in patients admitted to the ICU and periodically once a week. For this purpose, a pharyngeal and an axillary-rectal sample are collected and cultured in CHROMagar^TM^ Candida (Becton, Dickinson and Company, Franklin Lakes, NJ, USA). Plates are incubated at 37 °C for 48 h, and lectures are done at 24 and 48 h.

All patients with positive blood cultures for *C. auris* since October 2017, when the first episode was detected, to June 2020 were included. Blood samples were processed according to our routine laboratory procedure. Briefly, blood samples are incubated in BD Bactec^TM^ FX (Becton, Dickinson and Company, Franklin Lakes, NJ, USA) for 5 days or 14 days if fungi blood infection was suspected. When the sample flags positive, and the Gram stain reveals the presence of yeasts, subculture is performed in Sabouraud-Chloramphenicol and CHROMagar^TM^ Candida. In this medium, *C. auris* grows in a non-specific colour of white, beige or pink, and these colonies are further identified.

*Candida* isolates from both blood samples and surveillance samples are further identified by matrix-assisted laser desorption/ionisation (MALDI-TOF; Bruker, Billerica, MA, USA). Susceptibility testing is carried out for candidaemia isolates by broth microdilution through Sensititre^TM^ YestOne YO10 (Thermo Fisher Scientic, Waltham, MA, USA) for antifungals micafungin, caspofungin, anidulafungin, flucytosine, voriconazole, itraconazole, fluconazole and amphotericin B. Lastly, 15 isolates were tested through the new version of Sensititre^TM^ (Sensititre^TM^ YeastOne ITAMYUCC), which replaces flucytosine with isavuconazole. Tentative breakpoints from CDC were used to describe the susceptibility of the isolates to fluconazole (≥32 µg/mL), anidulafungin (≥4 µg/mL), caspofungin (≥2 µg/mL), micafungin (≥4 µg/mL) and amphotericin B (≥2 µg/mL) [28]. Only anidulafungin, micafungin, caspofungin and amphotericin B were reported in all isolates because the other antifungals were not routinely used in the treatment of *C. auris* infections. Isavuconazole was tested since it was available for use in the hospital. Fluconazole is also tested as a complementary method for identification since all isolates are resistant to it. Only the first isolate from every patient is included in the analysis.

Demographic and relevant clinical data were retrospectively collected via chart review, including comorbidities, underlying diseases, risk factors, duration of hospitalisation, duration of stay in the ICU before the candidaemia episode, previous antibiotic treatment, previous antifungal treatment, therapeutic measures and clinical outcome. Recurrence of candidaemia was defined as the case when a positive blood culture was obtained after a negative one. Persistence of candidaemia was defined as the case when a positive culture was obtained after 7 days of adequate antifungal treatment.

## 5. Conclusions

The reported outbreak in our setting is one of the most important globally, with prolonged transmission over two and a half years. Our experience, as well as the experience of other hospitals reported in the literature, show that *C. auris* can persist and efficiently spread in hospital environments, being difficult to eradicate. Instauration of infection control policies and periodic screening for colonisation are essential to control an outbreak caused by *C. auris*. Candidaemia is one of the most worrisome conditions caused by this multidrug-resistant yeast because it usually affects critical patients. Its multidrug resistance makes it even more difficult to manage these infections, especially since *C. auris* has potential to become more resistant in the course of a treatment with antifungal drugs. Therefore, patients with antifungal treatment for *C. auris* should be monitored closely and susceptibility testing should always be performed.

## Figures and Tables

**Table 1:** Epidemiological and clinical characteristics, antifungal treatment and outcome of *C. auris* candidaemia.

Characteristics	N = 47
Age, mean (SD)	61 (16.3)
Male	35 (74.5%)
APACHE II, mean (SD)	15.1 (7.0)
Candida score, mean (SD)	1.48 (1.18)
No risk	9 (19.2%)
Low risk	23 (48.9%)
High risk	15 (31.9%)
Charlson comorbidity index, mean (SD)	3.0 (2.1)
*Underlying disease*	
Abdominal surgery	14 (29.8%)
Digestive disease, not surgical	9 (19.1%)
Cardiovascular disease	7 (14.9%)
Neurovascular disease	5 (10.6%)
COVID-19	5 (10.6%)
Respiratory disease, not COVID-19	3 (6.4%)
Otorhinolaringologic disease	2 (4.3%)
Politrauma	2 (4.3%)
*Risk factors*	
Diabetes	13 (27.7%)
Malignancy	12 (25.5%)
Immunosuppression	2 (4.3%)
Renal replacement therapy	8 (17.0%)
Total parenteral nutrition	7 (14.9%)
Sepsis	10 (21.3%)
Surgery (<30 days before candidaemia)	24 (51.1%)
Mechanical ventilation	29 (61.7%)
Central venous catheter	39 (83.0%)
Urinary catheter	38 (80.9%)
ICU stay more than two weeks	33 (70.2%)
Previous antibiotic treatment	43 (91.5%)
Days administered:	
<7 days	3 (6.4%)
7 to 14 days	13 (27.7%)
>15 days	27 (57.4%)
Carbapenem use	25 (53.2%)
Previous antifungal treatment (administered for more than 5 days)	14 (29.8%)
Fluconazole	2 (4.3%)
Voriconazole	1 (2.1%)
Echinocandin	11 (23.4%)
*Therapeutic measures*	
Antifungal treatment	44 (93.6%)
Echinocandin in monotherapy	22 (46.8%)
Echinocandin plus Amphotericin B	12 (25.6%)
Echinocandin plus Isavuconazole	10 (21.3%)
Other measures	
Central venous catheter removal	33 (70.2%)
Urinary catheter replacement or removal	3 (6.4%)
*Outcome*	
30-day mortality	11 (23.4%)
*Complications*	
Endophtalmitis	2 (4.3%)
Recurrence of candidaemia	7 (14.9%)
Persistence of candidaemia	6 (12.8%)

**Table 2:** Antifungal susceptibility of *C. auris* isolates from blood cultures.

Antifungal	FLZ	AFG	MCF	CFG	AMB	PSC	VRC	ITC	5FC	ISA
**Isolates tested**	47	47	47	47	47	30	36	28	19	15
**Range**	256	0.06–0.25	0.03–0.5	0.03–0.25	0.125–1	0.015–0.5	0.5–8	0.06–0.5	0.06–0.25	0.03–0.5
**MIC_50_**	256	0.125	0.06	0.125	0.5	0.125	4	0.25	0.125	0.25
**MIC_90_**	256	0.25	0.125	0.25	1	0.25	8	0.5	0.25	0.5
**GM**	256	0.144	0.068	0.102	0.606	0.123	2.939	0.269	0.110	0.170

5FC, flucytosine; AFG, anidulafungin; AMB, amphotericin B; CFG, caspofungin; FLZ, fluconazole; GM, geometric mean MIC; ISA, isavuconazole; ITC, itraconazole; MFG, micafungin; MIC50 and MIC90, minimum inhibitory concentration required to inhibit the growth of 50% and 90% of the isolates, respectively; PSC, posaconazole; VRC, voriconazole.
